# Structure-guided affinity maturation of a novel human antibody targeting the SARS-CoV-2 nucleocapsid protein

**DOI:** 10.1038/s41598-022-12242-0

**Published:** 2022-05-19

**Authors:** Zhihong Wang, Naijing Hu, Yangyihua Zhou, Ning Shi, Beifen Shen, Longlong Luo, Jiannan Feng

**Affiliations:** State Key Laboratory of Toxicology and Medical Countermeasures, Beijing Institute of Pharmacology and Toxicology, Beijing, China

**Keywords:** Computational biology and bioinformatics, Drug discovery, Structural biology

## Abstract

The continuous mutation of SARS-CoV-2 has presented enormous challenges to global pandemic prevention and control. Recent studies have shown evidence that the genome sequence of SARS-CoV-2 nucleocapsid proteins is relatively conserved, and their biological functions are being confirmed. There is increasing evidence that the N protein will not only provide a specific diagnostic marker but also become an effective treatment target. In this study, 2G4, which specifically recognizes the N protein, was identified by screening a human phage display library. Based on the computer-guided homology modelling and molecular docking method used, the 3-D structures for the 2G4 scFv fragment (VH-linker-VL structure, with (G_4_S)_3_ as the linker peptide in the model), SARS-CoV-2 N protein and its complex were modelled and optimized with a suitable force field. The binding mode and key residues of the 2G4 and N protein interaction were predicted, and three mutant antibodies (named 2G4-M1, 2G4-M2 and 2G4-M3) with higher affinity were designed theoretically. Using directed point mutant technology, the three mutant antibodies were prepared, and their affinity was tested. Their affinity constants of approximately 0.19 nM (2G4-M1), 0.019 nM (2G4-M2) and 0.075 nM (2G4-M3) were at least one order of magnitude lower than that of the parent antibody (3 nM; 2G4, parent antibody), as determined using a biolayer interferometry (BLI) assay. It is expected that high-affinity candidates will be used for diagnosis and even as potential therapeutic drugs for the SARS-CoV-2 pandemic.

## Introduction

The spread of coronavirus disease 2019 (COVID-19) is a global health emergency affecting the entire world. As of 1 April 2022, the WHO has reported more than 474 million confirmed cases of COVID-19, including over 6 million confirmed deaths worldwide (https://covid19.who.int/). This has caused tremendous economic and social disruptions to countries worldwide. Through research and extensive vaccination with blockbuster vaccines (such as the US Pfizer/Germany BioNTech mRNA vaccine^1,2^, the University of Oxford and AstraZeneca-codeveloped recombinant adenovirus vaccine^3^, the Chinese inactivated vaccine^4,5^ and recombinant adenovirus vaccine^6^), the global pandemic has been effectively controlled. However, the frequent emergence of SARS-CoV-2 spike protein mutations has resulted in novel mutant strains (such as European variant D614G, British strain B.1.1.7, South African strain B.1.351, Indian strain B.1.617 and the omicron variant) with more aggressive infectivity and increased virulency^7^, posing new challenges to vaccine research and the development of drugs worldwide.

SARS-CoV-2 belongs to the genus β coronavirus. It is a single-stranded, enveloped, positive-stranded RNA with round or oval viral particles 60-140 nm in diameter^8^ and is generally polymorphic. The structural proteins of SARS-CoV-2 are composed of Spike protein (S), Envelope protein (E), Membrane protein (M) and Nucleocapsid protein (N)^9^. Among the four structural proteins, S protein binds to the receptor and promotes entry of the virus genetic material into the host cell^10^. M or E protein plays a certain role in assembly of the virion^11^. N protein is mostly used as a specific marker for the early detection of SARS-CoV-2^12^ or SARS^13^. According to the literature^14^, N protein, the most abundant and conserved coronavirus protein, is necessary for viral RNA replication and transcription, with three conserved domains, namely, the N-terminal domain (NTD), central linker (CL), and C-terminal domain (CTD). To date, many researchers have studied the structure and self-assembly characteristics of the N protein^15–17^. The X-ray crystal structures of the NTD^18^ and CTD^19,20^ of the first known N protein have also been solved. SARS-CoV-2 variants have also accumulated some mutations in the N protein. Gajendra Kumar Azad's research on the N protein of the Indian mutant strain pointed out mutations at sites 92, 152 and 156 in the NTD and that mutations at positions 344, 348 and 362 in the CTD might alter the secondary structure^21^. The study of N protein mutation sites might be of great significance for the screening of N protein-targeted drugs and the study of drug resistance.

Compared with the S protein, the SARS-CoV-2 N protein possesses a lower mutation rate and higher copy number and is the current marker for SARS-CoV-2 detection^22^. With continuous in-depth research on the N protein, increasing evidence indicates that the N protein might be a potential drug target^23–25^. During SARS-CoV-2 infection, the N protein is also highly immunogenic, similar to the S protein, and can also induce a protective immune response^26–28^. Zeng et al. found IgG, IgA and IgM antibodies against N protein in the serum of recovered patients, indicating the importance of N protein in diagnosis and the immune response^29^. Perna et al. also revealed the association of serum levels of N protein with disease severity, inflammation and progression in hospitalized patients^30^. Moreover, it has been shown that the highly pathogenic coronavirus N protein exacerbates lung injury through mannose (mannan)-binding lectin (MBL)-associated serine protease 2 (MASP-2)-mediated complement activation in a mouse model^31^. Furthermore, it was also confirmed that an anti-N protein antibody could inhibit excessive activation of complement^32^. Although the current treatments targeting the N protein are still controversial, with extensive research on N protein, an increasing number of researchers believe that N protein will become a drug target for the treatment of SARS-CoV-2^33–35^. At present, it has been confirmed that in the detection of SARS-CoV-2, high-affinity antibodies against N protein can effectively improve the sensitivity of detection, which is of great significance for the monitoring of the COVID-19 pandemic.

As progress is made in structural biology, bioinformatics, and computational biology, computer-aided antibody modification and de novo designs have become widely used. The emergence of virtual library designs for drug discovery eliminates the need for much in vitro screening work and improves the success rate of antibody reconstruction^36,37^. Our team has reported multiple studies about computer-aided antibody drug design and obtained candidate antibodies with better drug properties^38–40^. Moreover, we also successfully designed high-affinity receptor fusion proteins based on wild-type receptors with enhanced biological activity^41^.

In this study, a novel antibody, 2G4, targeting the SARS-CoV-2 N protein was identified by screening a phage display library. The affinity of 2G4 reached the nanomolar level, which is equivalent to that of the isolated fully human anti-N protein antibody nCoV396^32^. To further improve the affinity of the anti-N protein antibody (2G4), we analysed the 3-D theoretical structures of the antigen (i.e., N protein) and antibody (i.e., 2G4) using molecular simulation and docking methods. Based on the prediction results, three high-affinity antibody mutants were designed, namely, 2G4-M1, 2G4-M2 and 2G4-M3. The antibody mutants were prepared with a eukaryotic expression system, and the related affinities were determined by a biolayer interferometry assay. Ultimately, the affinity of the 2G4 mutants was increased markedly. The binding activity order was 2G4-M2 > 2G4-M1 > 2G4-M3 > 2G4. Our research not only provides a new method for antibody affinity maturation based on computer modelling but also provides three high-affinity scFv fragments targeting the SARS-CoV-2 N protein.

## Results

### Initial N protein antibody screening and generation of antibodies

**Figure 1:**
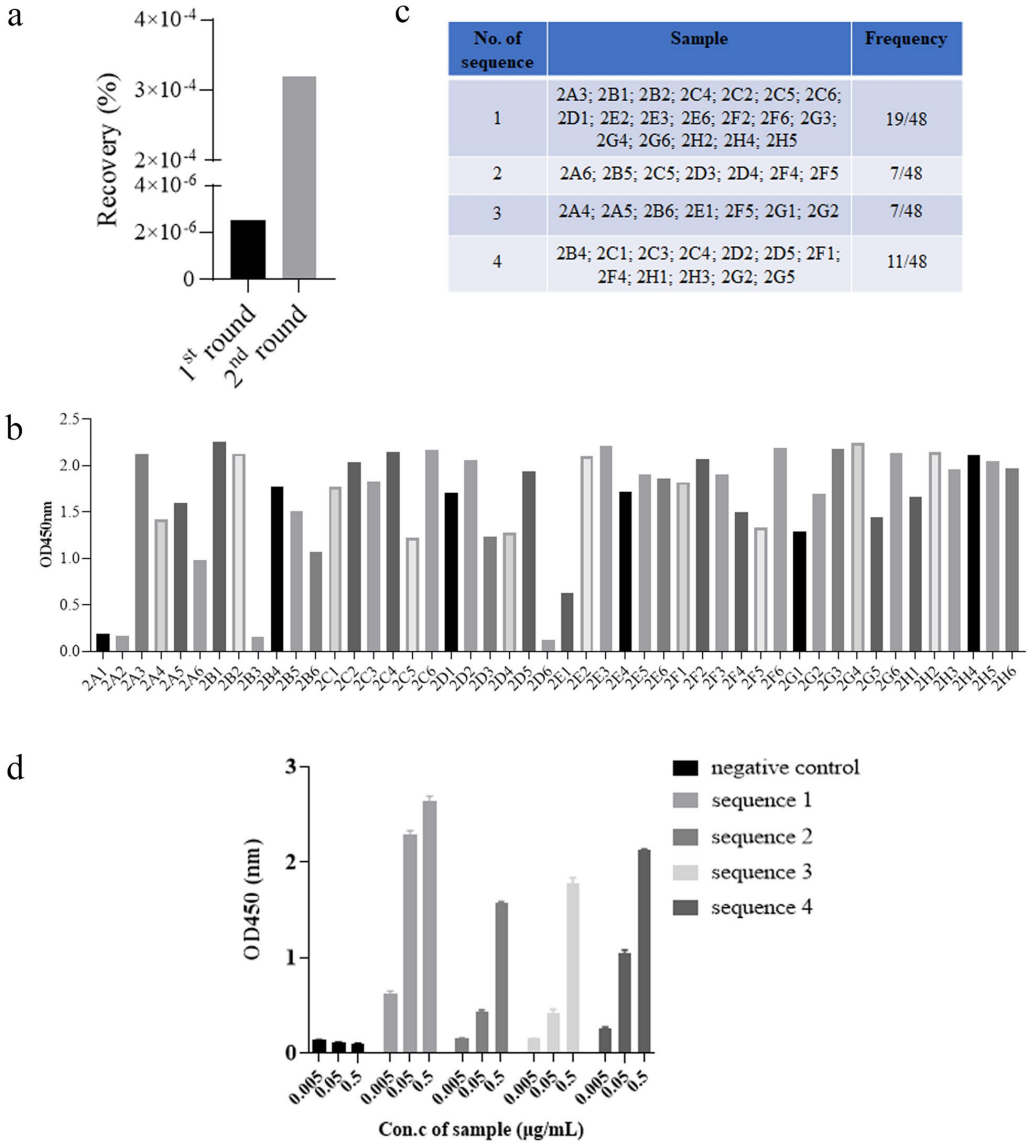
Screening of anti-SARS-CoV-2 nucleocapsid protein clones. (**a**) Comparison of the recovery ratios in different rounds of phage biopanning. (**b**) Phage ELISA analysis of 48 clones picked from the 2nd round of panning. (**c**) Frequency analysis of the amino acid sequences of phage panning positive clones. (**d**) Determination of the binding activity of the scFv fragments to N protein by ELISA.

Two rounds of biopanning were performed with enrichment of clones against N protein. The panning strategy is shown in Table 1. Successful enrichment of the binders was evidenced by the apparent increase in the recovery rate (output phages/input phages) after two rounds of panning (Fig. 1a). Next, we randomly selected 48 clones from the second panning output pools and prepared the phage supernatants. The subsequent ELISA results confirmed that the supernatant of the clones obtained after two rounds of panning could bind to the SARS-CoV-2 N protein (Fig. 1b). Sequence analysis (Fig. 1c) showed that a total of 4 sequences were successfully screened, among which the best binding activity was sequence 1 with the highest frequency, indicating efficient enrichment for N protein binders during selection. The four genes were then cloned into the expression vector PB513B-1 in an scFv format and transiently expressed by using the HEK293 expression system. The ELISA results (Fig. 1d) suggested that sequence 1 had the best binding activity with SARS-CoV-2 N proteins and could specifically bind to N protein in a concentration-dependent manner. We refer to sequence 1 as “2G4”. Overall, 2G4 exhibited binding activity to N protein and was selected as the candidate for further optimization.

**Table 1:** Panning strategy for isolating specific antibodies against the SARS-CoV-2 N protein.

Round	Coating antigen concentration (μg)	Input (cfu)	Blocking buffer	Washing times of PBST
1st	10	1 × 10^12^	4% skim milk	15
2nd	2	5 × 10^11^	4% skim milk	25

### 3-D theoretical structures of the 2G4 scFv fragment and complex conformation of the 2G4 scFv fragment and SARS-CoV-2 N protein

**Figure 2:**
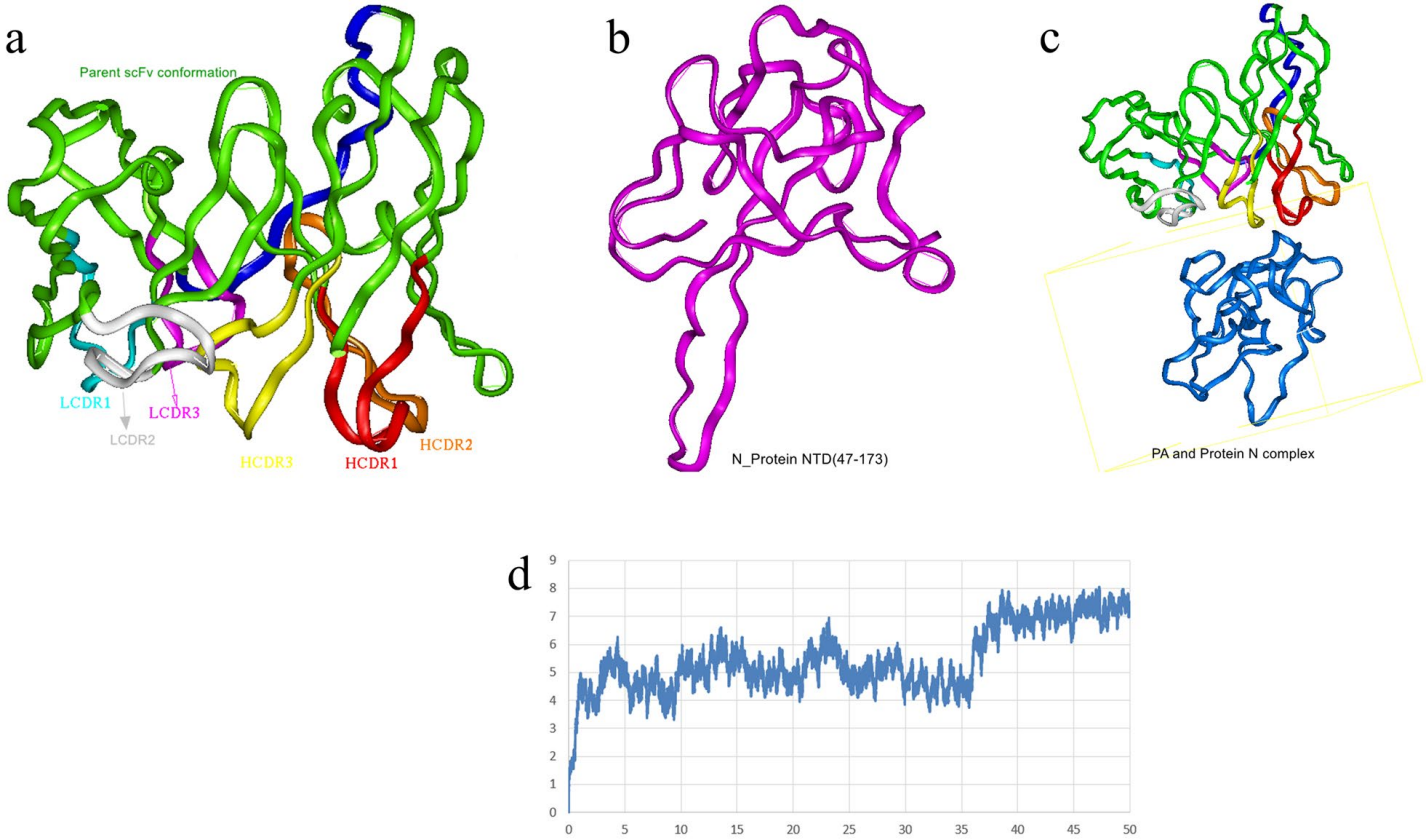
The 3-D modelling structures of the 2G4 scFv fragment (**a**), N protein N-terminus (**b**), and 2G4 scFv-N protein complex (**c**); the ribbon denotes the main chain carbon atom orientation of the proteins. (**a**) The green ribbon denotes the orientation of the 2G4 scFv fragment, the red ribbon denotes HCDR1, the orange denotes HCDR2, the yellow denotes HCDR3, the light blue denotes LCDR1, the white denotes LCDR2, the pink denotes LCDR3, and the deep blue denotes the linker. (**b**) The pink ribbon denotes the N-terminus of the N protein. (**b**) The green ribbon denotes the 2G4 scFv fragment, and the blue ribbon denotes the N-terminus of the N protein. (**d**) The RMSD calculation during the 50 ns MD simulation of the 2G4 scFv fragment and N protein (the X-axis is optimization time and Y-axis is RMSD (angstroms)).

The best match model (PDB code: 5jyl^42^) among 20 potential models was selected by processing with the BlastP program (http://www.ncbi.nlm.nih.gov/) and choosing from the PDB database. The alignment score between the model (PDB: 5jyl) and the 2G4 scFv fragment was 67.92%, and the theoretical structure of the 2G4 scFv fragment is shown in Fig. 2a. After 10,000 steps of minimization, the optimized structure of the 2G4 scFv was evaluated using a Ramachandran plot, and the assignment of the whole heavy atoms of the 2G4 scFv was in the credible range. Furthermore, the crystal structure of the SARS-CoV-2 nucleocapsid protein N-terminus (PDB code: 6m3m^18^) was chosen as a model, and the N-terminal theoretical structure of the N protein was obtained, as shown in Fig. 2b. Using the docking module in InsightII 2000 software and considering the interaction binding energy, 6 potential binding complex structures were chosen for minimization. The potential binding energy and binding mode are shown in Table S1. After 20,000 steps of optimization, the complex structure with the lowest binding energy and the most residues in the 2G4 scFv CDRs that attended to binding was chosen. The 3-D complex structure of the 2G4 scFv fragment and N-terminal N protein is shown in Fig. 2c. To deeply understand the stability of the complex structure of the 2G4 scFv fragment and N-protein, 50 ns molecular dynamics (MD) simulation was performed using the Discovey_3 module in Insight II 2000 software. The root mean square distance (RMSD) was calculated as shown in Fig. 2d. The results showed that the theoretical structure of the 2G4 scFv and N protein was stable.

### Key amino acids in the 2G4 scFv fragment identified by the N protein

**Figure 3:**
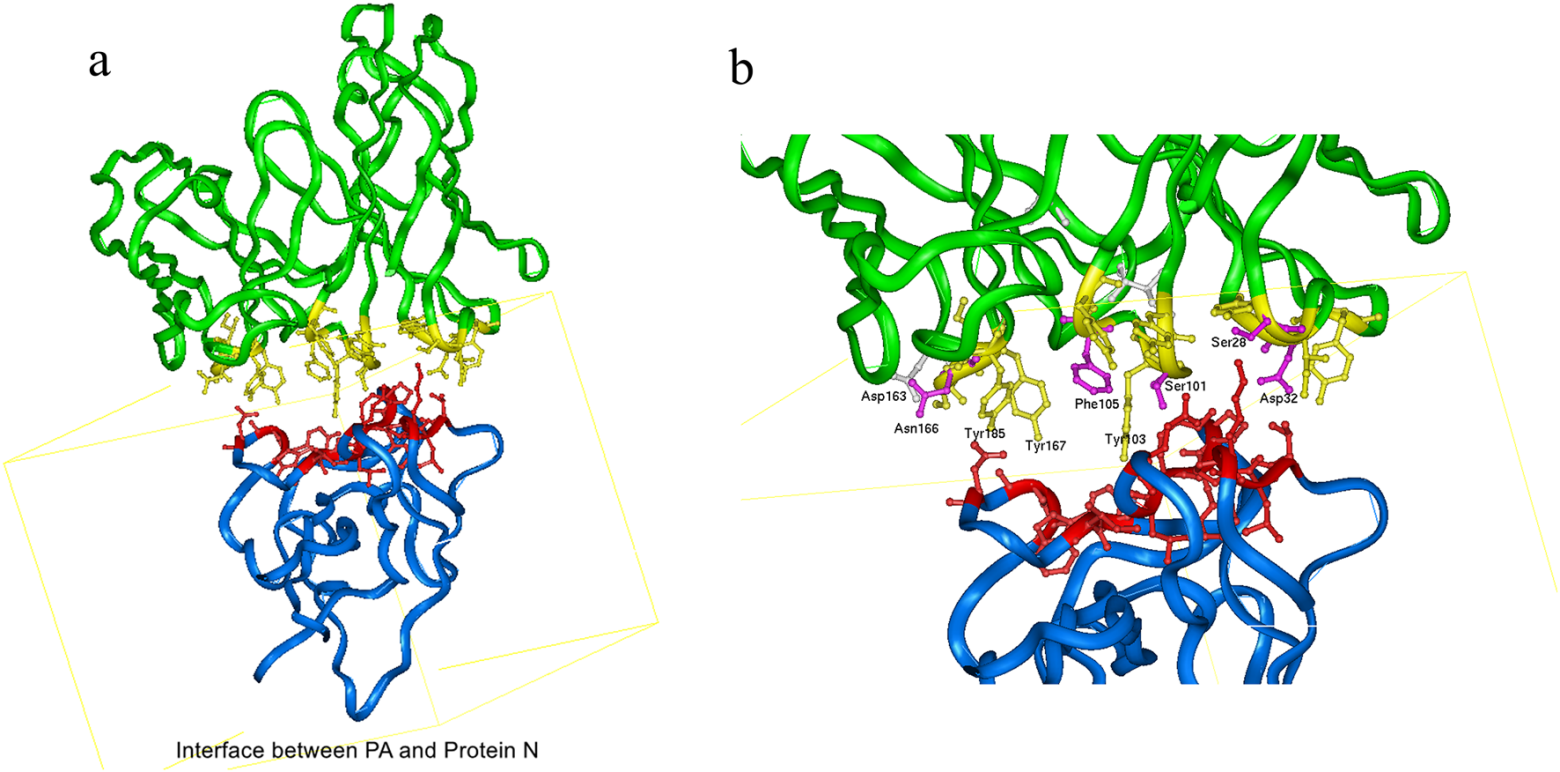
Analysis of important amino acid residues of 2G4 that interact with N protein. (**a**) The whole binding mode between the 2G4 scFv and N protein. The green ribbon denotes the 2G4 scFv fragment, and the blue ribbon denotes the N-terminus of the N protein. The yellow ball and stick denote the heavy atoms of the key residues in the 2G4 scFv, and red denotes the important residues in N protein. (**b**) The local binding mode between 2G4 and N protein. The red ball and stick denote the key amino acids in N protein, and the key amino acid residues are marked as name, position and heavy atom orientation.

According to the 3-D theoretical complex structure of the 2G4 scFv fragment and N proteins shown in Fig. 2c, the key amino acids in the 2G4 scFv identified by N proteins were predicted using the distance geometry method. The distribution of key residues was located in the complex structure, as shown in Fig. 3a. Based on the van der Waals interaction and coulombic binding activity, the interaction distance between key amino acid residues of the parent proteins (i.e., 2G4 scFv and N proteins) was defined as 6A. To deeply understand the binding between the 2G4 scFv fragment and N protein, the local map of the binding between the 2G4 scFv and N protein is shown in Fig. 3b. The key amino acid residues Ser^30^ and Asp^32^ (located in H_CDR1), Ser^101^ and Phe^105^ (located in H_CDR3), Asp^163^ and Asn^166^ (located in L_CDR1), and Ile^229^ and Ser^232^ (located in L_CDR3) were shown to bind to the N protein through van der Waals interactions (i.e., Phe^105^ and Ile^229^), hydrogen bonding (i.e., Ser^30^, Ser^101^, Ser^232^ and Asn1^66^) and electrostatic interactions (i.e., Asp^163^ and Asp^32^). In detail, the binding distance of the key residues between the 2G4 scFv fragment and N protein is shown in Table S2.

### Novel 2G4 scFv affinity maturation analysis and in silico design

**Figure 4:**
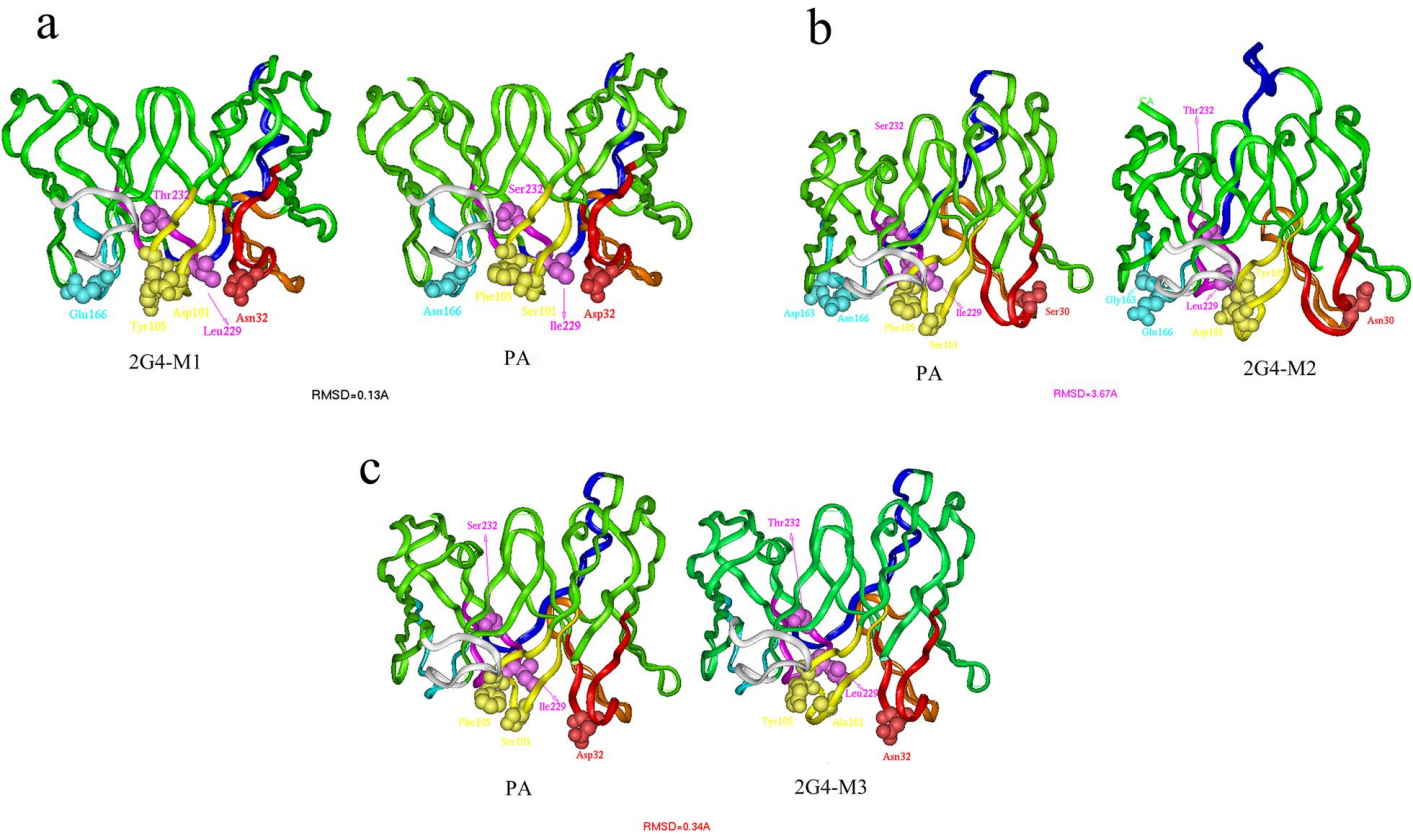
Conformation comparison of the 2G4 scFv fragment and the three mutants. The orientation of the parent (2G4 scFv) and mutants after structural superposition and translation. (**a**) The 3-D theoretical ribbon structures of 2G4-M1 and 2G4. (**b**) The 3-D theoretical ribbon structures of 2G4-M2 and 2G4. (**c**) The 3-D theoretical ribbon structures of 2G4-M3 and 2G4.

As shown in Fig. 3 and Table 2, the binding activity between the 2G4 scFv fragment HCDR1 and N protein was based on a polar interaction but not electrostatic activity, and the binding mode between the 2G4 scFv fragment HCDR3 and LCDR1 and N protein was based mainly on an electrostatic interaction. Considering the binding mode and key amino acid residue location of the 2G4 scFv fragment and based on the rule “maintain the potential epitopes, possible 3-D structure and surroundings”, three mutants of 2G4 scFv named 2G4-M1, 2G4-M2, and 2G4-M3 were designed. The complementarity-determining region (CDR) sequence alignment is shown in Table 2, and the corresponding mutants are marked in bold. It was shown that the point mutant retained the property of the parent or adopted a better binding mode, as mentioned above. Then, the 3-D structures of the mutants and the parent 2G4 were modelled and aligned using superimposition methods. The RMSD of the main chain carbon atoms was calculated, and the 3-D models are shown in Fig. 4a-c. The results show that the three mutants maintained the 3-D main chain conformation of the parent 2G4 scFv fragment, which implies that they might recognize a similar epitope as the 2G4 scFv fragment. Furthermore, by using the binding free energy calculation method, the binding energy between the scFv and N protein was calculated and compared with that of the parent antibody 2G4 scFv fragment, as shown in Table 3. The results showed that the three mutants possessed higher binding activity than the parent antibody 2G4, and the theoretical binding order was 2G4-M2 > 2G4-M1 > 2G4-M3 > 2G4.

**Table 2:** Amino acid sequences of the CDRs of 2G4 and mutants.

	CDR1		CDR2		CDR3	
	Light chain	Heavy chain	Light chain	Heavy chain	Light chain	Heavy chain
2G4-M1	SASQDIS**E**YLN	GYSISS**N**Y	YTSSLHS	SYSGS	QQYSK**L**PY**T**	GY**D**GYL**Y**YFDY
2G4-M2	SASQ**G**IS**E**YLN	GYSI**N**SDY	YTSSLHS	SYSGS	QQYSK**L**PY**T**	GY**D**GYL**Y**YFDY
2G4-M3	SASQDISNYLN	GYSISS**N**Y	YTSSLHS	SYSGS	QQYSK**L**PY**T**	GY**A**GYL**Y**YFDY
2G4	SASQDISNYLN	GYSISSDY	YTSSLHS	SYSGS	QQYSKIPYS	GYSGYLFYFDY

**Table 3:** The binding energy (kJ/mol) between the scFv and N protein.

Antibody	2G4	2G4-M1	2G4-M2	2G4-M3
Van der Waals	− 78.21	− 125.73	− 152.24	− 90.87
Electronic	− 40.46	− 64.08	− 39.29	− 55.59
Total	− 118.67	− 189.81	− 191.53	− 145.46
No. of intermolecular hydrogen bond	2	4	4	4

### In vivo characterization of the antibody by computational affinity maturation design

**Figure 5:**
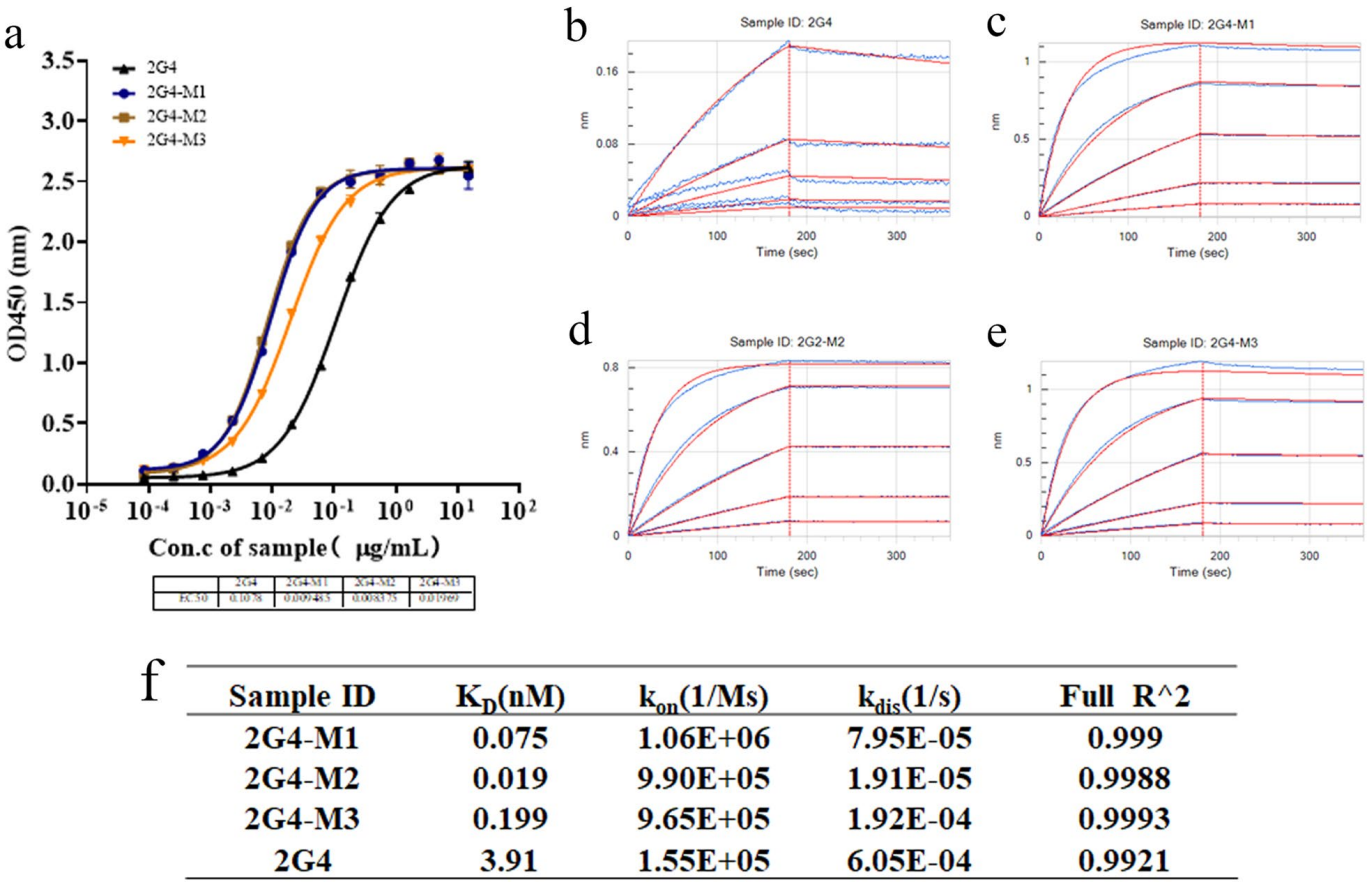
Characterization and comparison of anti-SARS-CoV-2 nucleocapsid protein mutant antibodies. (**a**) The antibody binding activity to the SARS-CoV-2 N protein was detected by an ELISA. (**b**–**f**) The binding kinetics of antibodies to the SARS-CoV-2 N protein were detected by BLI. Experiments were independently repeated at least three times, and one representative experiment is shown.

Based on the theoretical analysis and design and considering codon adaptation and RNA secondary structure, the mutant amino acid residues were reverse translated and expressed by using the HEK293 expression system, and the antibodies purified with protein A were evaluated using SDS–PAGE and HPLC. The results (Fig. S1A,B) showed that compared with the parent antibody, the mutant antibodies could be expressed without significantly changing the purity. In addition, the binding activity of the three mutant antibodies to the N protein was evaluated using an ELISA. The results, shown in Fig. 5a, indicate that the binding activity of the antibodies improved in the following order: 2G4-M2 > 2G4-M1 > 2G4-M3 > 2G4, which is consistent with the theoretical prediction. Next, BLI, a label-free technology for the development of antibodies and drugs that realizes affinity detection with high sensitivity^43^, was used. The association/dissociation curves are shown in Fig. 5b–e. Compared with that of 2G4, the affinity of 2G4-M3 to the N protein increased by 1 order of magnitude, that of 2G4-M1 increased ~ 20 times, and that of 2G4-M2 increased even further by 2 orders of magnitude. The experimental parameters are shown in Fig. 5f. The experimental results were in agreement with the theoretical predictions, indicating that the computer-aided mutant design was successful.

### Comparison of mutant antibodies recognizing core epitopes on SARS-CoV-2 N protein

**Figure 6:**
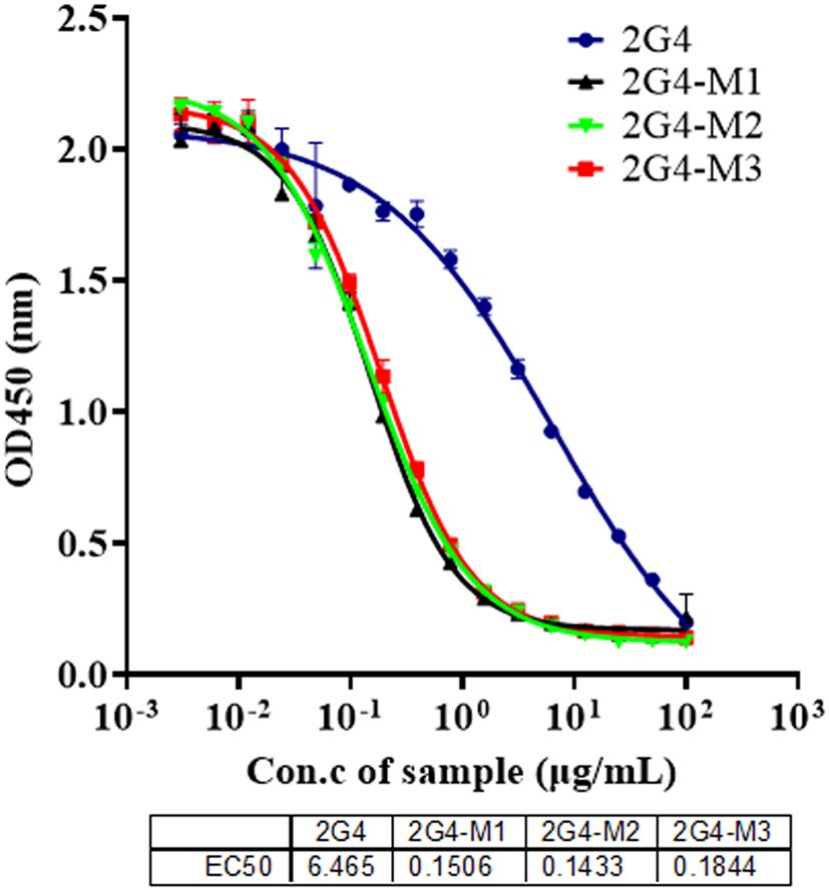
Confirmation of N protein epitope drift by competitive ELISA. A representative experiment out of two independent experiments is shown.

Given that the computer theoretical design may cause the occurrence of epitope drift, we decided to study whether the epitope of the N protein recognized by the three antibodies is the same as that recognized by 2G4. The result of a competitive ELISA is shown in Fig. 6. The three optimized anti-N protein antibodies were able to more effectively compete with 2G4 in binding to the N protein, indicating that there was no significant epitope drift among the three mutant antibodies.

Together, the overall data from the experiments show that the 3 optimized antibodies had better binding activity and affinity to the N protein and that there was no obvious drift in the recognition epitope. This result proves that the theoretical in vitro affinity maturation method is feasible and efficient and can effectively achieve antibody affinity maturation.

## Discussion

In December 2020, mutant strains of SARS-CoV-2 appeared in the United Kingdom, and their infectivity was 1.7 times greater than that of conventional virus strains. In April 2021, another mutant strain caused an outbreak of COVID-19 in India, causing great tension for global pandemic prevention. Since vaccines cannot achieve complete protection and the effect of vaccines is even more compromised for people with weak or impaired immune systems, the development of effective drugs for the treatment of COVID-19 is still imperative.

With the continuous emergence of mutant strains, the neutralizing titres of many targeted S protein therapeutic antibodies have decreased^44^. In stark contrast, the N protein genome sequence is conserved, and there have been reports confirming that anti-N protein antibodies play a vital role in inhibiting excessive activation of complement in COVID-19 treatment. Some researchers also pointed out that blocking the SARS-CoV-2 N protein is expected to be a treatment strategy to prevent the ongoing COVID-19 pandemic^45^. As virological research intensifies, an increasing number of researchers are carrying out research on the N protein^14,46,47^, and drugs specifically targeting the N protein have made promising progress in treatment^48,49^. As mentioned above, nCoV396 has been described as inhibiting the hyperactivation of complement^32^. Therefore, nCoV396 may be effective in reducing the mortality of patients with severe COVID-19. More importantly, the N protein is a key marker for SARS-CoV-2 detection, and affinity is usually positively correlated with detection sensitivity, which indicates that our higher-affinity antibodies might have greater potential in the detection of SARS-CoV-2. Although our work has not verified the therapeutic effect of the three antibodies, we hope to provide references and research tools for N protein-related research in the future.

The successful completion of the Human Genome Project has provided researchers with a large number of drug target molecules. Research on target molecules at the gene level and cell level is currently a hot area in drug development. In addition, CAD accelerates the speed and success rate of drug development, and computer homology modelling and molecular docking technology have improved the success rate of the design of some macromolecular drugs (typically antibody drugs). In recent years, artificial intelligence (AI) technology has become a hot topic for future drug development. For example, computer deep learning could be used to build a massive virtual antibody library and realize short-term (perhaps 2-3 days) virtual screening for unknown targets (such as a potentially more contagious variant of the coronavirus in the future) through supercomputing and molecular simulation docking. The rapid screening of antibody drugs will be a revolutionary breakthrough in the antibody drug industry. The emergence of this concept will disrupt the basic mode of traditional antibody drug screening and become a more efficient and rational antibody drug research and development platform. In this article, we successfully used computers to carry out in vitro affinity maturation of antibodies. Our research experiences lay the foundation for future artificial intelligence-based antibody design.

In conclusion, our data predicted 3 antibodies that have mature affinity for the N protein. Compared with that of 2G4, their affinity was significantly improved, and the recognized epitope did not show any obvious drift. We have carried out experimental verification of the computational data. Although the therapeutic effects have not been confirmed, it can effectively improve the sensitivity of theoretical SARS-CoV-2 detection. We hope that this work may provide a better choice for the diagnosis and treatment of COVID-19 and contribute to the early end of the COVID-19 pandemic.

## Materials and methods

### Initial screening of anti-SARS-CoV-2 N protein antibodies by phage display library

To screen for anti-SARS-CoV-2 N protein antibodies, a naïve phage library^40^ was used to isolate antibody clones binding to the N protein. Phage display screening was mainly carried out with the conventional method. The experimental procedures can be simply summarized as “adsorption”, “elution”, and “amplification”, and the input phages and output phages were calculated. In brief, the first round of biopanning for the enrichment of N protein binders was performed using 10 µg of N protein (Novoprotein, cat. DRA31) coated into the immune tube overnight at 4 °C. Then, 1 mL of 4% skim milk was used to block the immune tube at room temperature for 1 h. Meanwhile, the naïve phage library was preincubated with 500 μL of 4% skim milk for 1 h at room temperature. Following this, the pretreated library was added to the tube at room temperature for 1 h. Unbound phages were washed away using 0.1% PBST (Tween-20, v/v%). Subsequently, 500 μL of Gly-HCl (0.1 M, pH 2.2) was added and incubated for 10 min at room temperature to elute the bound phages, and 66.6 μL of Tris-HCl (1 M, pH 9.2) was added to neutralize the elution. Finally, 10 µL of elution solution was used to test the phage titre, and the remainder was amplified with logarithmic TG1 for the following panning. Two rounds of panning were performed to enrich the N protein-specific antibody binders. After two rounds of screening, the clones were picked out from the 2nd round output, and phage supernatants were prepared for further study.

### Phage ELISA

The 96-well enzyme-labelled array plates were coated with 100 μL per well of 1 μg/mL N protein diluted in carbonate buffer (0.05 M sodium carbonate-sodium bicarbonate buffer, pH 9.6), incubated overnight at 4 °C, and then blocked with 200 μL per well 4% skim milk for 1 h at 37 °C. Next, 100 μL of phage supernatants from the picked clones were added to the plates for 1 h at 37 °C. Then, a 1:6,000 dilution of anti-M13 antibody (HRP) (Sino Biological Inc., cat. 11973-MM05T-H) was prepared in PBS, and 100 μL of the secondary antibody was incubated for 45 min at room temperature. The plates were developed with a tetramethylbenzidine (TMB) solution (100 μL). The reaction was stopped after 3 min by adding 100 μL of 2 N H_2_SO_4_. The absorbance at 450 nm was determined for each well by a Molecular Devices Spectra Max 190.

### Computer-guided homology modelling and molecular docking

Based on the amino acid sequences of the variable domain of the 2G4 heavy and light chains, the 3-D theoretical structures of the 2G4 VH and VL fragments were constructed using a computer-guided homology modelling method. The homology modelling procedure was carried out as follows: (1) the amino acid residues of the 2G4 VH and VL fragments were aligned with the PDB database, and the best model was chosen; (2) the conserved region and loop domain were determined using the homology module of Insight II 2000 software; (3) the corresponding coordinates of the conserved region were assigned as a model, and the coordinates of the loop domain were modelled using ab initio modelling; (4) the intramolecular disulfide bond was built based on the antibody variable Kabat rule; and (5) the N-terminus and C-terminus were refined based on the homology module. Based on the 3-D predicted structure of the 2G4 VH and VL fragments, the 3-D structure of the 2G4 scFv fragment ((G_4_S)_3_ was chosen as the linker peptide) was modelled Considering the advantage of the CVFF forcefield^50,51^, the 3-D structure of the 2G4 scFv fragment was optimized with energy minimization under the CVFF forcefield. The final optimized 3-D structure of the 2G4 scFv fragment was evaluated using a Ramachandran plot.

In addition, the 3-D structure of the N-terminus of the SARS-CoV-2 N protein was obtained (the original structure was 6m3m from the PDB database) and optimized with the CVFF forcefield. The 3-D complex structure of the 2G4 scFv fragment and N-terminus of the N protein was constructed using the rigid molecular docking method of Insight II 2000 software. The docking procedure and parameters were assigned as follows: (1) the docking grid was created based on the 3-D structure of the SARS-CoV-2 N protein as the default value, and the cut-off was designated 8.0 angstroms; (2) using the grid assignment and the 2G4 scFv movement around the N-protein, the binding energy was calculated, and the complex structure with the lowest energy was chosen as the potential binding mode. To evaluate the binding mode from the docking module of Insight II 2000 software, AutoDock (https://autodock.scripps.edu/download-autodock4/) and free edition UCSF Chimaera were used to deeply evaluate the interaction between the 2G4 scFv fragment and N-protein.

With the determined 3-D structure of the 2G4 scFv fragment and N-protein, 50-ns molecular dynamics were performed with the Discovery_3 module of Insight II 2000 software. All calculations were performed using Insight II 2000 software (MSI Co., San Diego) with an IBM workstation.

### In silico mutagenesis for affinity maturation of 2G4

According to the theoretical 3-D complex structure of the 2G4 scFv fragment and N-terminus of the N-protein, the binding mode and key residues were determined. Given the physicochemical property and possible secondary structure of the amino acid residues and abiding by the rule “maintain the potential epitope, possible 3-D structure and surroundings”, the three mutant antibodies 2G4-M1, 2G4-M2 and 2G4-M3 were designed. On the basis of the same structural modelling and energy optimization methods mentioned above, the binding energy was calculated and compared with the binding energy of the parent antibody 2G4 and the N protein.

### Expression of anti-N protein antibodies in HEK293 cells and purification

The antibody gene was fused with the Fc of human IgG1 in the form of scFv-Fc and synthesized by GENEWIZ, and then the genes were subcloned into the expression vector PB513B-1. Then, the expression vectors were transfected into HEK293 cells using *JetPRIME* (Polyplus Transfection, cat. 114-15), which were cultured for 72 h. The culture supernatants were collected and filtered through a 0.45 μm filter. The antibodies were purified by protein A affinity chromatography using a HiTrap MabSelect SuRe column (GE Healthcare) in an AKTA Purifier purification system (GE Healthcare). Finally, all the antibodies were concentrated by ultrafiltration with an Amicon Ultra-15 Centrifugal Filter Unit with a 50-kDa molecular weight cut-off and analysed by SDS-PAGE and HPLC.

### Indirect ELISA

The 96-well enzyme-labelled array plates were coated with 1 μg/mL SARS-CoV-2 N protein (100 μL) diluted in carbonate buffer and incubated overnight at 4 °C. The plates were subsequently blocked with 4% skim milk for 1 h at 37 °C. The antibodies were diluted with PBS to 15 μg/mL. On this basis, a total of 12 concentrations were obtained by threefold serial dilution and added into the appropriate wells, and then the plate was incubated for 1 h at 37 °C. Next, the plates were incubated with an HRP-conjugated goat anti-human IgG (H + L) secondary antibody (Invitrogen, cat. A18805) at room temperature for 45 min. The plates were developed with TMB solution, and the reaction was stopped after 3 min by adding 2 N H_2_SO_4_. The absorbance at 450 nm was determined for each well using a Molecular Devices Spectra Max 190.

### Affinity determination

To assay the affinity of antibodies to N protein, biolayer interferometry was performed on a FortéBio Octet 96e instrument. The 6 × His-tagged N protein was diluted to 10 μg/mL with running buffer (PBST, PBS containing 0.02% (v/v) Tween-20) and tethered to a Ni–NTA-coated biosensor. The antibodies were diluted to the corresponding concentrations (33.33 nM, 11.11 nM, 3.70 nM, 1.23 nM, 0.41 nM and 0 nM) in running buffer. Then, the biosensor was dipped into a solution containing the antibodies for 180 s, followed by dissociation for 180 s. The biosensor regeneration was performed with a 10 mM glycine HCl (pH 1.7) solution and pulsed 3 times for 5 s each. The data were analyzed using FortéBio Data Analysis 11.1 (Sartorius, FortéBio®). The resulting data were fitted into a 1:1 binding model from which K_on_ and K_off_ values were obtained, and then the equilibrium dissociation constant KD values were calculated.

### Competitive ELISA

The enzyme-labelled array plate was coated with N protein (1 μg/mL) overnight at 4 °C and then blocked with 4% skim milk for 1 h at 37 °C. The antibodies were diluted with biotinylated 2G4 (1 μg/mL) to 100 μg/mL. On this basis, a total of 12 concentrations were then obtained by twofold serial dilution and added into the appropriate wells, and then the plate was incubated for 1 h at 37 °C. Streptavidin-HRP (Thermo Scientific, cat. 21130) was added to the wells, and the plates were incubated at room temperature for 45 min. Absorbance at 450 nm was determined for each well using a Molecular Devices Spectra Max 190.

## Supplementary Information

Supplementary Figure S1.

Supplementary Table S1.

Supplementary Table S2.

## Data Availability

The original contributions presented in the study are included in the article/Supplementary Material, and further inquiries can be directed to the corresponding authors.
