# Supplementary figures and images for: Structure-guided affinity maturation of a novel human antibody targeting the SARS-CoV-2 nucleocapsid protein
Source: Sci Rep. 2022 May 19;12:8469. doi: 10.1038/s41598-022-12242-0 (PMC9118815; doi:10.1038/s41598-022-12242-0)

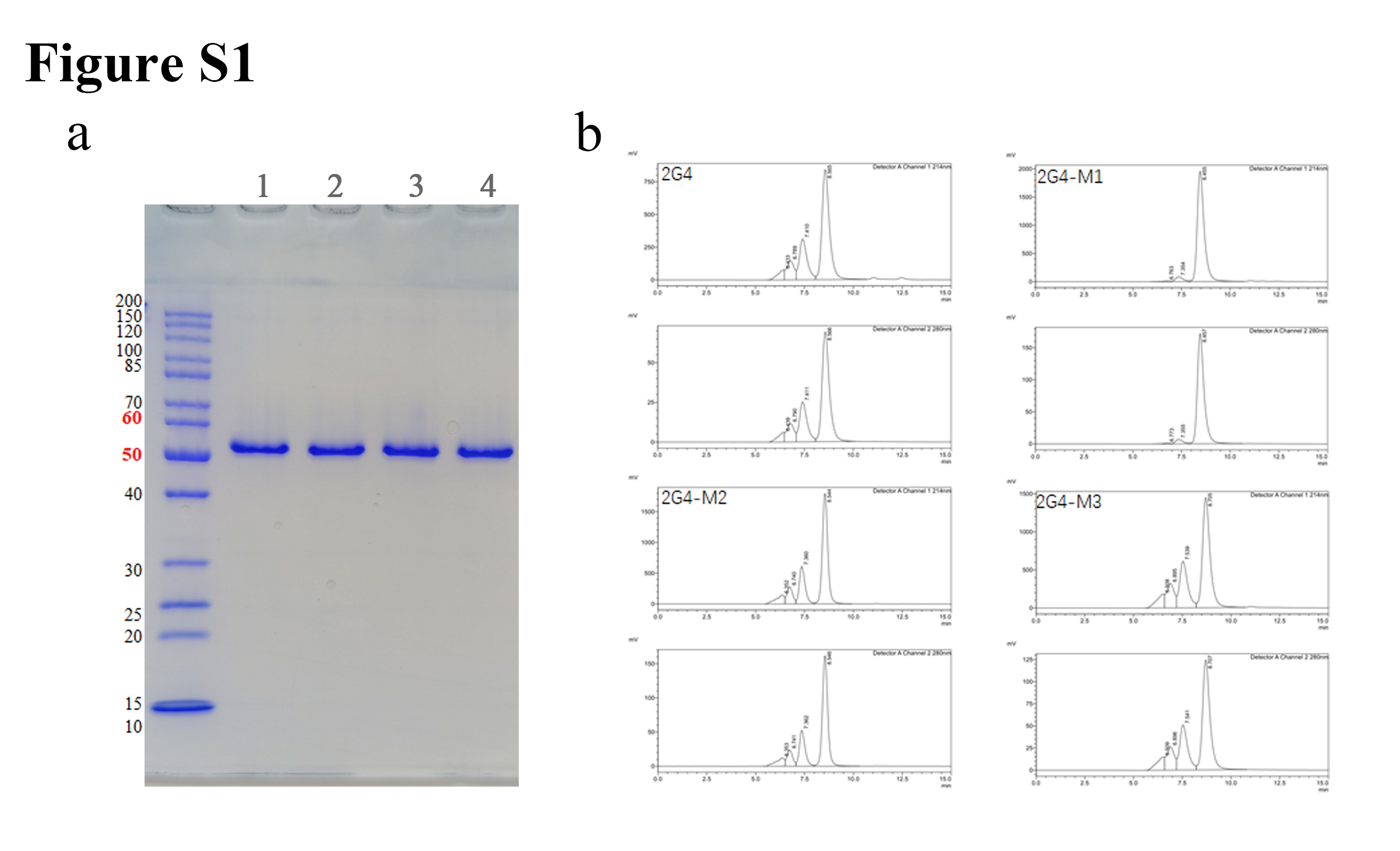

Supplement: Supplementary file 1 — Supplementary Figure S1. [file 41598_2022_12242_MOESM1_ESM.tif]
