# Supplementary material for: Structure-guided affinity maturation of a novel human antibody targeting the SARS-CoV-2 nucleocapsid protein
Source: Sci Rep. 2022 May 19;12:8469. doi: 10.1038/s41598-022-12242-0 (PMC9118815; doi:10.1038/s41598-022-12242-0)
Supplement: Supplementary file 2 — Supplementary Table S1. [file 41598_2022_12242_MOESM2_ESM.docx]

**TABLE S1**. Potential binding complex obtained based on DOCK module in Insight II 2000 software

| Binding Model | Potential binding residues of N protein | Binding energy (kcal/mol) |
| --- | --- | --- |
| 1 | Glu^62^Leu^64^Lys^65^Phe^66^Pro^67^, Tyr^123^Gly^124^, Asn^126^Ile^131^Trp132, Thr^166^Leu^167^Pro^168^Lys^169^ | -28.38 |
| 2 | Lys^61^Glu^62^Asp^63^Arg^68^, Asp^81^Asp^82^Gln^83^ | -24.71 |
| 3 | Tyr^88^Tyr^87^Arg^88^, Lys^100^Lys^102^Asp^103^, Tyr^111^Tyr^112^Thr^115^ | -21.09 |
| 4 | Tyr^123^Gly^124^Ala^125^Asn^126^Lys^127^Asp^128^ | -18.72 |
| 5 | Asn^140^Thr^141^Lys^143^ASP^144^His^145^Thr^148^ | -20.13 |
| 6 | Asn^153^Asn^154^Ile^157^Gln^163^Thr^165^Thr^166^ | -19.34 |
