# Supplementary material for: Structure-guided affinity maturation of a novel human antibody targeting the SARS-CoV-2 nucleocapsid protein
Source: Sci Rep. 2022 May 19;12:8469. doi: 10.1038/s41598-022-12242-0 (PMC9118815; doi:10.1038/s41598-022-12242-0)
Supplement: Supplementary file 3 — Supplementary Table S2. [file 41598_2022_12242_MOESM3_ESM.docx]

**TABLE S2.** The binding mode between 2G4 scFv fragment and N protein

| Binding Mode | 2G4 scFv Fragment | N protein | Distance  (angstrom) |
| --- | --- | --- | --- |
| Hydrogen Bond | Ser^30^ | Lys^169^ | 3.84 |
|  | Ser^232^ | Asn^126^ | 6.28 |
|  | Asn^166^ | Asn^126^ | 6.50 |
|  | Ser^101^ | Glu^62^ | 4.93 |
| Electrostatic interaction | Asp^32^ | Lys^169^ | 3.52 |
|  | Asp^163^ | Lys^169^ | 5.28 |
| Van der Waals interaction | Phe^105^ | Ile^131^ | 4.92 |
